# Frequency of Acute Kidney Injury and Association With Mortality Among Extremely Preterm Infants

**DOI:** 10.1001/jamanetworkopen.2022.46327

**Published:** 2022-12-13

**Authors:** Khyzer B. Aziz, Eric M. Schles, Kartikeya Makker, James L. Wynn

**Affiliations:** 1 Department of Pediatrics, Johns Hopkins University, Baltimore, Maryland; 2 Johns Hopkins Technology and Innovation Center, Johns Hopkins University, Baltimore, Maryland; 3 Department of Pediatrics, University of Florida, Gainesville; 4 Department of Pathology, Immunology, and Laboratory Medicine, University of Florida, Gainesville

## Abstract

**Importance:**

Neonatal acute kidney injury (AKI) is common and associated with morbidity and mortality. The temporal relationship between AKI and critical illness, as well as the frequency of AKI definition components (urine output and serum creatinine [sCr] concentration change), are unknown in extremely low-birth-weight (ELBW) (<1000 g), extremely preterm (<29 weeks’ completed gestational age [GA]) infants.

**Objective:**

To measure the frequency of AKI from birth to death or discharge with attention to the definition components as well as the temporal relationship of AKI to critical illness and death.

**Design, Setting, and Participants:**

A single-center, multiyear, retrospective cohort study was conducted at an academic level IV neonatal intensive care unit between January 1, 2012, and January 1, 2020. Participants included inborn ELBW and infants at 22 to 28 weeks’ completed GA with confirmed congenital anomalies who survived 12 hours or more.

**Exposures:**

Extremely preterm birth and ELBW.

**Main Outcomes and Measures:**

The primary outcome was AKI frequency. The timing, severity, and criteria for AKI were measured. The temporal relationship between AKI, organ dysfunction, and outcomes were quantified using odds ratios (ORs), logistic regression, and Shapley Additive Explanations. Acute kidney injury recognition, imaging, pediatric nephrology consultation, and follow-up were determined.

**Results:**

A total of 436 infants (52% male; 44% Black) met the inclusion criteria (median BW, 725 g; median GA, 25.7 wk). Acute kidney injury was common in the first week of life (44%), primarily based on the change in the sCr concentration criterion (88%), and negatively associated with GA (OR, 0.69; 95% CI, 0.60-0.78), but positively associated with antecedent critical illness (OR, 1.17; 95% CI, 1.12-1.23), severe intraventricular hemorrhage (OR, 1.86; 95% CI, 1.12-3.08), late-onset sepsis (OR, 1.03; 95% CI, 1.02-1.03), and mortality (OR, 2.77; 95% CI, 1.63-4.72). Acute kidney injury had negligible clinical contribution to death within the model (Shapley Additive Explanation, <0.5% change to outcome) relative to antecedent patient-concentration organ dysfunction (6%-15% change). Among infants with severe AKI, recognition (32%), nephrology inpatient consultation (16%), and outpatient follow-up (9%) were not common.

**Conclusions and Relevance:**

In this cohort study of ELBW infants, AKI was common in the first week of life, inversely associated with GA, and followed organ (primarily cardiovascular) dysfunction. Acute kidney injury considered as the primary pathway to mortality was rare, and amelioration of AKI to modify death was not well supported.

## Key Points

**Question:**

Is there an association between acute kidney injury (AKI), critical illness, and death in the extremely low-birth-weight preterm infant?

**Findings:**

In this cohort study of 436 extremely low-birth-weight infants, AKI was (1) inversely proportional to gestational age and birth weight, (2) primarily based on the change in the serum creatinine criterion, (3) preceded by organ dysfunction other than the kidney, and (4) showed a negligible clinical contribution to death in modeling.

**Meaning:**

The findings of this study suggest that AKI followed organ, primarily cardiovascular, dysfunction; amelioration of AKI to modify the outcome of death was not well supported.

## Teaser

This cohort study examines the frequency and markers of acute kidney injury in extremely low-birth-weight and extremely preterm infants.

## Introduction

Acute kidney injury (AKI) is routinely encountered in pediatric and adult intensive care units (ICUs), associated with adverse outcomes including mortality, and frequently and successfully mitigated by kidney replacement therapy.^1,2^ Patients with AKI have double the ICU costs compared with those without AKI.^3^ Among patients admitted to the neonatal ICU (NICU), AKI frequency ranges from 18% to 70%^4^ and is bimodal with peaks in term infants with hypoxic-ischemic encephalopathy or congenital surgical disease and in extremely low-birth-weight (ELBW) infants (birth weight <1000 g).^5,6,7,8,9^ Short-term mortality risk and long-term kidney complications, including hypertension, are associated with neonatal AKI.^10^

Neonatal AKI is commonly defined using age-modified Kidney Disease: Improving Global Outcomes (KDIGO) criteria wherein the severity is determined by the magnitude of changes in serum creatinine (sCr^Δ^) concentration or urine output (UOP).^11^ Using these criteria, the prevalence and severity of AKI as well as the association of AKI with outcomes of interest in different neonatal populations and at different times after birth have been characterized.^10^ A secondary analysis of a large cohort of ELBWs, who have the greatest risk of AKI among NICU patients, showed that stage 3 AKI (most severe) may portend subsequent mortality based on sCr concentration measurements collected as part of standard clinical care.^5^ However, that study did not include longitudinal measures of organ dysfunction or shock. Thus, the longitudinal incidence of AKI from birth to death or discharge in this unique population, including the criterion and values that substantiated AKI, as well as the temporal relationship of AKI to neonatal severity of illness and adverse inhospital outcomes, remains unclear. Our objective was to comprehensively measure AKI from birth to death or discharge in a high-risk population with special attention to which KDIGO component verified AKI (UOP or sCr^Δ^ concentration). We describe clinical characteristics of patients who developed AKI, when AKI developed, the association between AKI and adverse inhospital outcomes, and the association of AKI with mortality and severity of illness metrics as measured by the neonatal Sequential Organ Failure Assessment (nSOFA) and vasoactive-inotropic score (VIS). In addition, we describe the number of patients with AKI documentation, imaging, pediatric nephrology consultation, and follow-up.

## Methods

This single-center, retrospective cohort study was approved by the University of Florida Institutional Review Board before data collection. This study was deemed exempt from patient consent by the University of Florida Institutional Review Board because it posed minimal risk and is secondary research use of identifiable private information for which consent is not required. An integrated data repository was created with all clinical data in the electronic health record for all ELBW infants and infants with less than 29 weeks’ completed gestation admitted to the University of Florida Health concentration IV NICU between January 1, 2012, and January 1, 2020. Infants born at outside hospitals, survived for less than 12 hours, had severe congenital anomalies, or completed less than 22 weeks’ gestation were excluded.^12,13^ Demographic variables and outcomes were defined as previously reported (eMethods in the Supplement).^13,14,15,16,17,18^ Maternal race was extracted from the electronic health record. Race was self-designated by the mother of the infant on admission. Racial and ethnic data were included given the known genetic variations in measurable kidney function parameters (Table 1; eTable 6 in the Supplement). The decision to group into Black, White, and other was based on the number of patients in these respective groups. For all deaths (all cause), detailed medical record review was performed to identify the primary contributors to death. Maximum VIS and nSOFA scores were calculated as previously described.^12,19^ The nSOFA includes respiratory (fraction of inspired oxygen, oxygen saturation as measured by pulse oximetry, and intubated status), cardiovascular (use of systemic steroids, qualitative vasoactive-inotropic drug exposure), and hematologic (platelet count) system parameters. The VIS was calculated as follows: dopamine dose (micrograms per kilograms per minute) + dobutamine dose (micrograms per kilograms per minute) + 100 × epinephrine dose (micrograms per kilograms per minute) + (10 × milrinone dose [micrograms per kilograms per minute] + 10 × vasopressin dose [milliunits per kilogram per minute] + 100 × norepinephrine dose [micrograms per kilograms per minute]). The nSOFA index, which represents the average weekly sum of nSOFA scores measured hourly during the first 28 or 60 days of life, were calculated.^13^ This report follows the Strengthening the Reporting of Observational Studies in Epidemiology (STROBE) reporting guideline for cohort studies (eMethods in the Supplement).

**Table 1.** Cohort Demographic Characteristics

Variable	In first week, No. (%)			*P* value
	No AKI (n = 244)	Stage 1 AKI (n = 134)	Stage 2-3 AKI (n = 58)	
**Maternal**				
Age, median (IQR), y	27 (23-33)	27 (22-32)	27 (21-34)	.68
Race and ethnicity				
Black	105 (43)	60 (45)	25 (43)	.77
White	106 (43)	60 (45)	23 (40)	
Other^a^	33 (14)	14 (10)	10 (17)	
Pregnancy-induced hypertension	85 (35)	30 (22)	19 (33)	.01
PPROM	83 (34)	30 (22)	16 (28)	.06
Preterm labor	147 (60)	94 (70)	32 (55)	.07
Antenatal corticosteroids^b^	229 (94)	127 (95)	53 (91)	.67
Vaginal delivery	74 (30)	50 (37)	25 (43)	.12
Chorioamnionitis^c^	116/241 (48)	70/132 (53)	31/56 (55)	.50
Abruption	36 (15)	16 (12)	6 (10)	.60
**Neonatal**				
GA, median (IQR)	26.1 (24.9-27.1)	25.6 (24.4-26.6)	24.1 (23.5-25.9)	<.001
BW, median (IQR)	785 (646-880)	706 (590-841)	584 (510-726)	<.001
SGA	30 (12)	19 (14)	14 (24)	.07
Sex				
Female	114 (47)	68 (51)	29 (50)	.73
Male	130 (53)	68 (51)	29 (50)	.85
5-min Apgar score	6 (4-8)	6 (4-7)	6 (4-7)	.23
Early sepsis	7 (3)	3 (2)	1 (2)	.86
SIP	12 (3)	12 (9)	6 (10)	.18
SIVH^d^	36/239 (15)	29/130 (22)	24/54 (44)	<.001
Prolonged early antibiotics	165 (68)	83 (62)	43 (74)	.23
Late sepsis^e^	56/233 (24)	36/122 (30)	13/43 (30)	.45
NEC	26 (11)	18 (13)	5 (9)	.57
ROP (any stage)^f^	131/216 (61)^g^	73/104 (70)	21/23 (91)	.007
SROP^f^	37/216 (17)^g^	30/104 (29)	9/23 (39)	.008
BPD (oxygen at 36 wk)^f^	122/216 (56)^g^	63/104 (61)	13/23 (57)	.78
BPD-DC with supplemental oxygen^f^	74/216 (34)^g^	43/104 (41)	8/23 (35)	.46
Death	27 (11)	30 (22)	35 (60)	<.001
Age at death, median (IQR), d	5 (2-21)	5 (3-17)	4 (3-11)	.74
Length of stay, median (IQR), d^f^	94 (79-114)	104 (85-123)	117 (89-129)	.01
Maximum nSOFA prior to first week of AKI diagnosis, median (IQR)	4 (2-8)	6 (2-10)	11 (6-3)	<.001
Maximum VIS prior to first week of AKI diagnosis, median (IQR)	0 (0-5)	3 (0-10)	12.5 (3-30)	<.001

Abbreviations: AKI, acute kidney injury; DC, discharged; BW, birth weight; GA, gestational age; NEC, necrotizing enterocolitis; nSOFA, neonatal Sequential Organ Failure Assessment; PPROM, preterm premature rupture of membranes; ROP, severe retinopathy of prematurity; SGA, small for GA; SIP, spontaneous intestinal perforation; SIVH, severe intraventricular hemorrhage (grade 3-4); SROP, severe ROP; VIS, vasoactive-inotropic score.

^a^ Other designations for race and ethnicity included American Indian (n = 1), multiracial (n = 13), unknown (n = 5), and other (not further defined by the patient) (n = 38).

^b^ Any exposure.

^c^ Missing data on 6 patients.

^d^ Missing data on 13 patients.

^e^ Among survivors for more than 72 hours.

^f^ Among survivors to discharge.

^g^ One infant was discharged to an outside hospital prior to completion of 36 weeks and was excluded from these calculations.

### Acute Kidney Injury

Acute kidney injury was defined using KDIGO criteria.^20^ Stage 1 AKI was an sCr concentration increase of 0.3 mg/dL within 48 hours or an sCr concentration increase of 1.5 to 1.9 × a reference sCr concentration* (*lowest prior sCr concentration for each patient) or UOP during 24 hours greater than 0.5 and less than or equal to 1 mL/kg/h. Stage 2 AKI was an sCr concentration increase of 2.0 to 2.9 × a reference sCr* or UOP during 24 hours greater than 0.3 and less than or equal to 0.5 mL/kg/h. Stage 3 AKI was an sCr concentration increase of greater than or equal to 3 × a reference sCr* or any sCr concentration greater than or equal to 2.5 mg/dL (to convert to micromoles per liter, multiply by 88.4) or UOP during 24 hours and less than or equal to 0.3 mL/kg/h. No patient in this cohort received dialysis. Acute kidney injury was considered present when either UOP or the sCr^Δ^ criterion was met. Stage 1 AKI was defined as mild (mAKI) and greater than or equal to stage 2 AKI was considered severe (sAKI). When both UOP and the sCr^Δ^ criterion for AKI were met, the highest stage of AKI was used for calculations. Acute kidney injury after the first week of life was categorized by the sCr^Δ^ criterion alone,^11^ because UOP beyond diaper counts is not routinely measured once intravenous fluids are stopped. Accordingly, we separated our analysis of AKI into the first week of life and after the first week of life based on the availability of UOP measures. Serum creatinine values reported as less than X were changed to a value of X for calculations (eg, <0.2 = 0.2). If there was more than 1 sCr concentration measurement per day, the greatest value was used for calculations. Daily UOP was used for calculations. The day of life (DOL)1 UOP was not used for DOL1 AKI calculations if the neonate was aged less than 8 hours at the time daily UOP was recorded.

### Statistical Analysis

Outcomes for regression analysis were death, death at DOL7 or less, and death at longer than DOL7. We used univariable regression for any AKI, sAKI (anytime), and sAKI (≤DOL7 and >DOL7) and adjusted the AKI-associated outcomes for relevant factors. In addition, a structural bayesian model and a logistic regression model were built to assess the interactions between the dependent variable and independent variables. Shapley Additive Explanations (SHAP) values were used to determine which variables were most important to the logistic regression models. The goal of SHAP is to explain the prediction of outcome by computing the contribution of each feature to the prediction. SHAP values can be interpreted as the contribution of a feature value to the difference between the actual prediction and the mean prediction. The SHAP value is the average marginal contribution of a feature value across all possible coalitions. Details for modeling are presented in the eMethods in the Supplement. The threshold for statistical significance was *P* < .05 for 2-sided tests. Analyses were performed using GraphPad Prism, version 9 (group comparisons and graph productions), R, version 3.6.2 calculation of hourly VIS/nSOFA from raw electronic health record data, Stata, version 15.2 (StataCorp LLC) for ORs in tables, or Python, version 3.7, for structural models and SHAP analysis.

## Results

### Patients

The 436 infants (52% male; 44% Black) who met our inclusion criteria (eFigure 1 in the Supplement) were categorized into 3 groups based on the presence of AKI in the first week of life for comparison of demographic characteristics and common outcomes. Among infants with first-week sAKI, gestational age and birth weight were lower, length of stay was longer, and severe intraventricular hemorrhage (SIVH), retinopathy of prematurity, severe retinopathy of prematurity, and death were more common than in those without AKI or with mAKI. Among nonsurvivors (death at any time; n = 92), first-week mAKI occurred in 30 infants (33%) and sAKI occurred in 35 (38%); 27 infants (29%) manifested no AKI. Among survivors (n = 344), first-week mAKI occurred in 104 infants (30%) and sAKI in occurred in 23 (7%); 217 infants (63%) manifested no first-week AKI. The median sCr measure per patient among all patients (n = 436) was 27 sCr (IQR, 16-41 sCr), the median sCr measure per patient for survivors (n = 344) was 30 sCr (IQR, 21-44 sCr), and the median sCr measure per patient for nonsurvivors (n = 92) was 10 sCr (IQR, 4-26 sCr).

### First Week of Life AKI

All included infants had 2 or more sCr measures and a daily UOP measured in the first week of life. There were 5144 measures of sCr concentrations in 436 patients in the first week of life. Urine output was recorded on each day alive for 436 infants (eFigure 2 in the Supplement). Acute kidney injury (any stage) occurred on each DOL in the first week (range, 13%-32%) and peaked on DOL2 (eFigure 3A in the Supplement). Mild AKI was the most common manifestation of AKI (DOL1-7 range, stage 1: 66.7%-79.8%, stage 2: 10.0%-20.8%, and stage 3: 4.5%-12.5%) (eFigure 3B in the Supplement). Among patients with AKI, daily UOP less than or equal to 1 mL/kg/h was uncommon (median, 16%; IQR, 6%-19%) compared with Cr^Δ^ greater than or equal to 0.3 mg/dL (53%; IQR, 40%-73%) or a greater than or equal to 1.5-fold Cr^Δ^ (88%; IQR, 78%-96%) (eFigure 3C in the Supplement).

The maximum sCr concentration was greater in infants with AKI on each day of the first week of life than those without AKI (eFigure 4A in the Supplement). Urine output less than or equal to 1 mL/kg/h as well as the extent of positive fluid balance between patients with and without AKI were manifest largely in the first 4 DOL (eFigure 4B and C, eTable 1 in the Supplement). An examination of the patient-concentration trajectory in the first week of life revealed AKI was concentrated in the smallest and least mature neonates (eFigure 5 in the Supplement). Neonates born at less than or equal to 24 weeks (n = 147) represented 45% of all first-week AKI (any stage) and 62% of all first-week sAKI.

### Association Between Organ Dysfunction and Shock to First Week of Life AKI

The maximum VIS and maximum nSOFA scores prior to diagnosis of the first episode of AKI were greater among infants with sAKI compared with those with mAKI or no AKI (Table 1). Among patients with first-week sAKI, 43 (74%) had vasoactive-inotropic drug exposure prior to the first episode of AKI. Clinical and laboratory characteristics of the 15 (stage 2: n = 12, stage 3: n = 3) patients with sAKI who had no prior vasoactive-inotropic drug exposure had an antecedent maximum nSOFA score greater than or equal to 4 (n = 7) alongside common surrogates of antecedent poor perfusion including placental abruption or absent end-diastolic flow (n = 3), 5-minute Apgar score less than or equal to 5 (n = 7), base deficit less than or equal to −10 (n = 6), or lactate concentration greater than or equal to 2 (n = 5).

### AKI After the First Week of Life

Among the 378 patients who survived beyond DOL7, 84 (22%) subsequently manifested mAKI, 59 (16%) developed sAKI (8 with sCr concentration ≥2.5 mg/dL), and 235 (62%) had no AKI (eFigure 6 in the Supplement). No AKI beyond DOL7 was most common in infants with no prior AKI (70%) but was similar among those with first-week mAKI (51%) and those with first-week sAKI (46%). Mild AKI beyond DOL7 was most prevalent among infants with first-week mAKI (28%) compared with no prior AKI (20%) or first-week sAKI (18%). Severe AKI after DOL7 was rare in infants with no prior AKI (10%) compared with first-week mAKI (22%) and those with first-week sAKI (36%). The age for mAKI after the first week (median, 24 days; IQR, 17-70 days) was similar to age for sAKI (DOL26; IQR, 17-79; *P* = .15). The absolute sCr measures (all) that qualified patients for mAKI were less than those (all) that qualified patients for sAKI (0.68 mg/dL; IQR, 0.32-1.08 mg/dL vs 1.23 mg/dL; IQR, 0.61-1.97 mg/dL; *P* < .001). Antecedent vasoactive-inotropic drug exposure occurred in 44 of 59 infants (75%) and late-onset sepsis or necrotizing enterocolitis occurred in 30 of 59 infants (51%). Peak sCr values in sAKI episodes after the first week of life were less than 1 mg/dL in 22 of 59 infants (37%) (1.3 mg/dL; IQR, 0.77-1.83 mg/dL). The nSOFA index over the first 28 days of life was higher among patients with sAKI (282; IQR, 108-513; *P* < .001 vs no AKI; *P* = .003 vs mAKI) compared with patients with mAKI (165; IQR, 36-394; *P* < .001 vs no AKI) or no AKI (56; IQR, 51-80). Similarly, the nSOFA index over the first 60 days of life was higher among patients with sAKI (441; IQR, 77-801; *P* < .001 vs no AKI; *P* = .002 vs mAKI) compared with patients with mAKI (107; IQR, 19-247; *P* < .001 vs no AKI) or no AKI (32; IQR, 3-106).

### Modeling

#### ORs for Neonatal Outcomes

The unadjusted odds of AKI or sAKI diminished with increasing GA and was increased among infants with SIVH and late-onset sepsis, and with respect to the maximum nSOFA or maximum VIS (Table 2). After adjustment for GA, the OR for SIVH to AKI or sAKI was no longer significant, whereas the maximum nSOFA, maximum VIS, and late-onset sepsis retained significant ORs for AKI or sAKI. Although the ORs for sAKI in the first week among infants with SIVH remained significant, the unadjusted OR (3.73; 95% CI, 2.04-6.80) decreased after adjustment for either GA (2.30; 95% CI, 1.19-4.47), maximum nSOFA (1.95; 95% CI, 1.01-3.78), or maximum VIS (2.42; 95% CI, 1.24-4.70) (eTable 2 in the Supplement). The odds of sAKI after DOL7 diminished with increasing GA and were increased among infants with late-onset sepsis and with respect to maximum VIS or maximum nSOFA. After adjustment for GA, late-onset sepsis, maximum nSOFA and maximum VIS retained significant ORs for sAKI after DOL7. To examine the association between AKI and death, unadjusted ORs for several clinical entities associated with death anytime, death at DOL7 or earlier, and death after DOL7 were calculated and adjusted for GA, AKI, or sAKI (Table 3; eTable 3 in the Supplement). The ORs for death for most conditions remained significant and were rarely and minimally reduced after adjustment for AKI or sAKI. Consistent with prior studies, AKI and sAKI were associated with a greater OR for death.^1,2,4,5,6,7,8,9,10,11,21^ After adjustment for GA only, the odds of any AKI contributing to death were no longer significant, but sAKI was associated with an OR of 2.08 (95% CI, 1.19-3.63; *P* = .01). Similar outcomes were found in analyses restricted to death timing. These data support the association of sAKI with death but do not address the temporal association or contribution of sAKI to death.

**Table 2.** ORs for AKI by Timing and Diagnoses

Variable	OR (95% CI)	*P* value	Adjusted (95% CI)					
			GA	*P* value	Maximum 28-d nSOFA	*P* value	Maximum VIS	*P* value
**AKI (anytime)**								
Sex	0.94 (0.64-1.38)	.75	0.90 (0.61-1.35)	.62	1.02 (0.68-1.53)	.92	0.96 (0.65-1.43)	.86
Male	1 [Reference]		1 [Reference]		1 [Reference]		1 [Reference]	
Racial or ethnic minority group^a^	0.87 (0.57-1.31)	.50	0.91 (0.60-1.41)	.70	0.80 (0.51-1.24)	.32	0.87 (0.57-1.33)	.53
White	1 [Reference]		1 [Reference]		1 [Reference]		1 [Reference]	
GA	0.69 (0.60-0.78)	<.001			0.81 (0.69-0.93)	.004	0.74 (0.65-0.85)	.005
Maximum VIS	1.05 (1.03-1.07)	<.001	1.03 (1.01-1.05)	.005	NA	NA	NA	NA
Maximum 28-d nSOFA	1.17 (1.12-1.23)	<.001	1.12 (1.07-1.19)	<.001	NA	NA	NA	NA
SIVH	1.86 (1.12-3.08)	.02	1.10 (0.63-1.90)	.76	1.10 (0.64-1.91)	.73	1.32 (0.77-2.25)	.32
SIP	2.34 (0.98-5.85)	.06	1.61 (0.65-3.97)	.30	1.41 (0.57-3.48)	.46	1.64 (0.67-4.04)	.28
NEC	1.61 (0.85-3.06)	.14	1.49 (0.77-2.89)	.2	1.27 (0.65-2.50)	.48	1.42 (0.74-2.75)	.30
Late-onset sepsis^b^	1.03 (1.02-1.03)	<.001	1.02 (1.01-1.03)	<.001	1.02 (1.01-1.04)	<.001	1.03 (1.02-1.04)	<.001
**sAKI (anytime)**								
Sex	1.20 (0.77-1.87)	.41	1.16 (0.74-1.84)	.51	1.34 (0.84-2.15)	.23	1.24 (0.79-1.97)	.35
Male	1 [Reference]	NA	1 [Reference]	NA	1 [Reference]	NA	1 [Reference]	NA
Racial or ethnic minority group^a^	0.79 (0.50-1.28)	.35	0.86 (0.52-1.40)	.54	0.73 (0.44-1.21)	.23	0.82 (0.50-1.35)	.44
White	1 [Reference]	NA	1 [Reference]		1 [Reference]		1 [Reference]	
GA	0.66 (0.57-0.77)	<.001	NA	NA	0.85 (0.71-1.00)	.05	0.74 (0.63-0.88)	<.001
Maximum VIS	1.04 (1.03-1.06)	<.001	1.03 (1.01-1.05)	<.001	NA	NA	NA	NA
Maximum 28-d nSOFA	1.24 (1.17-1.33)	<.001	1.21 (1.13-1.29)	<.001	NA	NA	NA	NA
SIVH	2.46 (1.48-4.08)	<.001	1.52 (0.87-2.65)	.14	1.35 (0.77-2.36)	.30	1.73 (0.99-2.99)	.05
SIP	1.38 (0.61-3.12)	.43	0.90 (0.38-2.11)	.82	0.73 (0.30-1.72)	.48	0.99 (0.49-2.02)	.99
NEC	1.16 (0.58-2.28)	.67	1.08 (0.54-2.17)	.83	0.80 (0.38-1.66)	.55	1.00 (0.47-1.97)	.99
Late-onset sepsis^b^	1.02 (1.01-1.02)	<.001	1.02 (1.01-1.03)	<.001	1.02 (1.01-1.03)	.001	1.02 (1.01-1.03)	<.001
**sAKI (>DOL7)**								
Sex	1.29 (0.74-2.25)	.38	1.18 (0.66-2.10)	.58	1.35 (0.75-2.41)	.32	1.31 (0.74-2.33)	.35
Male	1 [Reference]	NA	1 [Reference]	NA	1 [Reference]	NA	1 [Reference]	NA
Racial or ethnic minority group^a^	0.73 (0.40-1.34)	.31	0.86 (0.46-1.62)	.66)	0.68 (0.37-1.29)	.24	0.78 (0.42-1.44)	.43
White	1 [Reference]	NA	1 [Reference]	NA	1 [Reference]	NA	1 [Reference]	NA
GA	0.64 (0.52-0.78)	<.001			0.79 (0.64-0.98)	.04	0.70 (0.56-0.86)	<.001
Maximum VIS	1.05 (1.02-1.07)	<.001	1.03 (1.01-1.06)	.007	NA	NA	NA	NA
Maximum 28-d nSOFA	1.23 (1.14-1.33)	<.001	1.18 (1.09-1.29)	<.001	NA	NA	NA	NA
SIVH	1.79 (0.89-3.58)	.10	1.18 (0.55-2.45)	.67	1.03 (0.49-2.20)	.91	1.39 (0.67-2.89)	.38
SIP	1.91 (0.77-4.72)	.16	1.13 (0.43-2.93)	.81	0.90 (0.35-2.35)	.83	0.95 (0.33-2.68)	.92
NEC	1.56 (0.73-3.33)	.26	1.31 (0.59-2.87)	.50	0.95 (0.42-2.15)	.90	1.13 (0.50-2.54)	.78
Late-onset sepsis^b^	1.03 (1.02-1.04)	<.001	1.02 (1.01-1.03)	<.001	1.02 (1.01-1.02)	<.001	1.02 (1.01-1.03)	<.001

Abbreviations: AKI, acute kidney injury; DOL, day of life; GA, gestational age; NA, not applicable; NEC, necrotizing enterocolitis; nSOFA, neonatal Sequential Organ Failure Assessment; OR, odds ratio; sAKI, severe AKI; SIP, spontaneous intestinal perforation; SIVH, severe intraventricular hemorrhage; VIS, vasoactive-inotropic score.

^a^ The groups included Black and American Indian, multiracial, unknown, and other (not further defined by the patient).

^b^ Among survivors for more than 72 hours.

**Table 3.** ORs for Death

Variable	OR (95% CI)	*P* value	Adjusted (95% CI)					
			GA	*P* value	Any AKI	*P* value	Any sAKI	*P* value
**Death (anytime)**								
Sex	0.89 (0.56-1.41)	.62	0.77 (0.46-1.29)	.33	0.90 (0.56-1.44)	.66	0.84 (0.52-1.35)	.47
Male	1 [Reference]	NA	1 [Reference]	NA	NA	NA	1 [Reference]	NA
Racial or ethnic minority group^a^	1.04 (0.63-1.69)	.88	1.30 (0.75-2.27)	.36	1.08 (0.66-1.79)	.76	1.11 (0.68-1.84)	.68
White	1 [Reference]	NA	1 [Reference]	NA	NA	NA	1 [Reference]	NA
GA	0.44 (0.35-0.53)	<.001	NA	NA	0.45 (0.36-0.56)	<.001	0.46 (0.38-0.57)	<.001
Maximum VIS	1.20 (1.15-1.24)	<.001	1.18 (1.14-1.23)	<.001	1.20 (1.16-1.24)	<.001	1.20 (1.16-1.24)	<.001
Maximum 28-d nSOFA	1.90 (1.65-2.19)	<.001	1.82 (1.57-2.10)	<.001	1.90 (1.65-2.19)	<.001	1.89 (1.64-2.18)	<.001
SIVH	8.74 (5.07-15.04)	<.001	4.4 (2.42-8.01)	<.001	8.27 (4.75-14.38)	<.001	7.85 (4.48-13.72),	<.001
SIP	2.72 (1.26-5.86)	.01	1.43 (0.61-3.33)	.42	2.38 (1.09-5.22)	.03	2.68 (1.20-5.97)	.02
NEC	1.78 (0.92-3.42)	.09	1.83 (0.88-3.81)	.11	1.64 (0.82-3.19)	.15	1.78 (0.90-3.51)	.10
Any AKI	2.77 (1.63-4.72)	<.001	1.65 (0.92-2.97)	.10	NA	NA	NA	NA
Severe AKI	3.52 (2.15-5.76)	<.001	2.08 (1.19-3.63)	.01	NA	NA	NA	NA
**Death > DOL7**								
Sex	1.24 (0.62-2.51)	.54	1.19 (0.58-2.42)	.64	1.27 (0.62-2.60)	.51	1.19 (0.59-2.43)	.63
Male	1 [Reference]	NA	1 [Reference]	NA	NA	NA	1 [Reference]	NA
Racial or ethnic minority group^a^	0.64 (0.32-1.38)	.26	0.69 (0.32-1.50)	.36	0.67 (0.31-1.44)	.31	0.68 (0.31-1.48)	.33
White	1 [Reference]	NA	1 [Reference]	NA	NA	NA	1 [Reference]	NA
GA	0.64 (0.50-0.82)	<.001	NA	NA	0.68 (0.52-0.87)	.003	0.69 (0.53-0.88)	.005
Maximum VIS	1.05 (1.03-1.08)	<.001	1.07 (1.05-1.09)	<.001	1.05 (1.03-1.07)	<.001	1.05 (1.03-1.07)	<.001
Maximum nSOFA	2.10 (1.67-2.64)	<.001	2.13 (1.67-2.70)	<.001	2.09 (1.65-2.64)	<.001	2.14 (1.68-2.73)	<.001
SIVH	2.84 (1.34-5.99)	.006	1.63 (0.71-3.73)	.25	2.54 (1.19-5.41)	.02	2.42 (1.12-5.22)	.02
SIP	7.96 (3.35-18.88)	<.001	5.80 (2.37-14.21)	<.001	7.04 (2.92-16.95)	<.001	8.01 (3.28-19.54)	<.001
NEC	6.29 (2.91-13.61)	<.001	6.49 (2.91-14.43)	<.001	5.91 (2.69-12.95)	<.001	6.53 (2.94-14.46)	<.001
Any AKI	3.42 (1.39-8.44)	.008	2.50 (0.99-6.31)	.054	NA	NA	NA	NA
Severe AKI	3.13 (1.52-6.39)	.002	2.24 (1.05-4.74)	.04	NA	NA	NA	NA

Abbreviations: AKI, acute kidney injury; DOL, day of life; GA, gestational age; NA, not applicable; NEC, necrotizing enterocolitis; nSOFA, neonatal Sequential Organ Failure Assessment; OR, odds ratio; sAKI, severe AKI; SIP, spontaneous intestinal perforation; SIVH, severe intraventricular hemorrhage (grade 3-4); VIS, vasoactive-inotropic score.

^a^ The groups included Black and American Indian, multiracial, unknown, and other (not further defined by the patient).

#### Structural Models and SHAP Analysis for Neonatal Outcomes

**Figure 1.**
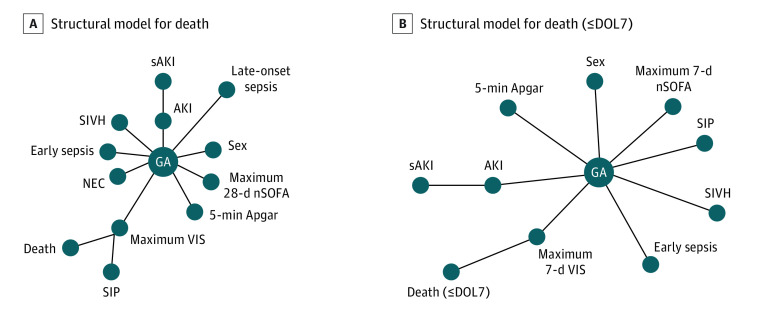
Structural Models and Shapley Additive Explanations (SHAP) Values A, Structural models illustrating relationships between individual clinical variables and outcomes (features) and death (primary node). Lines and circles represent undirected relationships among variables using the maximum likelihood estimation. B, Structural model for death as the primary mode. AKI indicates acute kidney injury; DOL7, day of life 7; GA, gestational age; NEC, necrotizing enterocolitis; nSOFA, Neonatal Sequential Organ Failure Assessment; sAKI, severe AKI; SIP, spontaneous intestinal perforation; SIVH, severe intraventricular hemorrhage; and VIS, vasoactive inotropic score.

**Figure 2.**
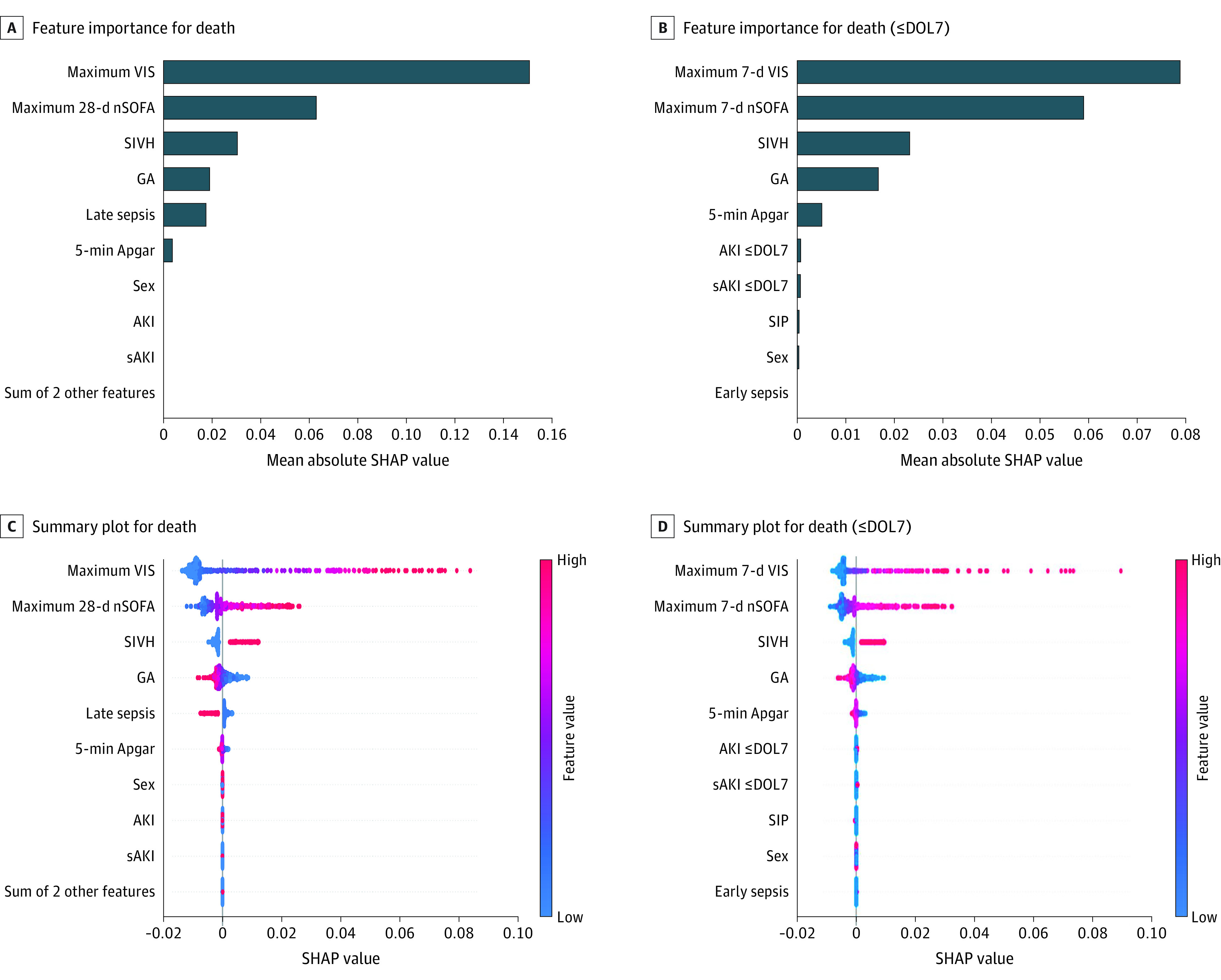
The Importance of Individual Variables, Effects on the Model, and Directionality of Effect A, Shapley Additive Explanations (SHAP) feature importance for death (anytime) as mean absolute SHAP values. B, SHAP feature importance for death (≤day of life 7 [DOL7]) as mean absolute SHAP values. C, SHAP summary plot for death (anytime). D, SHAP summary plot for death (≤DOL7). The higher the SHAP value of a feature, the higher the probability of death. A dot is created for each feature attribution value for the model of each patient, and thus 1 patient is allocated 1 dot on the line for each feature. Dots are colored according to the values of features for the respective patient and accumulate vertically to depict density. Red represents higher feature values and blue represents lower feature values. Sum of 2 other features (variables) includes spontaneous intestinal perforation and early-onset sepsis. AKI indicates acute kidney injury; DOL7, day of life 7; GA, gestational age; nSOFA, Neonatal Sequential Organ Failure Assessment; sAKI, severe AKI; SIVH, severe intraventricular hemorrhage; and VIS, vasoactive inotropic score.

The nondirectional maximum likelihood structural models for death anytime (Figure 1A), death at less than or equal to DOL7 (Figure 1B), and death after DOL7 (eFigure 7A in the Supplement) revealed that the AKI and sAKI tree structure was associated with lower GA but not death. In all structural models, dendritic branches showed associations of lower GA and adverse clinical outcomes (SIVH, necrotizing enterocolitis, length of stay, and early-onset sepsis). Acute kidney injury after DOL7 was associated with GA rather than history of AKI at less than or equal to DOL7 (eFigure 7A in the Supplement). Logistic regression modeling to assess the magnitude of importance for clinical variables showed greater GA had a negative association with death (contribution to survival) as expected (eFigure 8A in the Supplement). The maximum nSOFA up to 28 days had the greatest contribution to death followed by the maximum VIS (eFigure 8C and D in the Supplement). Acute kidney injury and sAKI clustered primarily at a score of 0, suggesting a minimal contribution of these entities on the outcome of death (eFigure 8E and F in the Supplement). The model average precision, recall, and F1 score (the harmonic mean of precision and recall in machine learning models) were very good to excellent (eFigure 8G-I in the Supplement). SHAP values revealed that the importance of variables in the logistic regression model (Figure 2A-C; eFigure 7B in the Supplement). Maximum VIS was most important (largest absolute SHAP value) and changed the predicted outcome of the model by 15% (Figure 2; eFigure 7C in the Supplement). The predicted outcome was changed by 6% for maximum nSOFA up to 28 days and GA by 2%. SHAP summary plots (eFigure 8C-F, eFigure 7C in the Supplement), which combined variable importance with variable outcomes for the model, showed maximum VIS was most important for mortality followed by maximum nSOFA up to 28 days, SIVH, and GA. Acute kidney injury and sAKI had negligible importance and no association with death within the model (SHAP value clustering at 0) compared with patient-concentration severity of illness as measured by maximum nSOFA and maximum VIS. Among the 92 deaths, 2 had documentation that AKI was a primary contributor (eTable 4 in the Supplement).

#### Recognition of sAKI

Among patients with sAKI at any time (n = 105), 41 (39%) died. Documentation of any AKI-like problem in the electronic health record (including notes [history and physical, daily, pharmacokinetic, consultant, nurse, respiratory therapist, and death summaries] written by the team caring for the infant, pathology reports, problem lists, *ICD* coding, and laboratory test results) or as an indication for radiologist interpretation of imaging (inclusive of all potentially associated with AKI, including renal/kidney failure, renal/kidney dysfunction, renal/kidney injury, oliguria/low UOP, elevated Cr, and uremia) was found for 34 of 105 infants (32%). The timing of potentially nephrotoxic medication doses, as well as all instances in which AKI occurred according to the sCr^Δ^ criterion during the hospitalization, were collated (eFigure 9 in the Supplement). Hypertension was documented in 14% of patients with sAKI, 13% with mAKI, and 8% with no AKI. Among infants with sAKI, none received nicardipine during the hospitalization and 3 of 64 (5%) among survivors received hydralazine or amlodipine at any time. Imaging (abdominal or retroperitoneal ultrasonography) was performed at any time and for any indication (not kidney specific) in 48 of 105 infants (46%). Pediatric nephrology consultation was performed in 17 of 105 infants (16%) for any indication (not AKI specific), and 6 of 64 (9%) survivors had nephrology outpatient follow-up.

## Discussion

In this cohort of extremely premature ELBW infants examined at high resolution from birth to death or discharge, AKI was (1) common, especially in the first week of life; (2) largely substantiated by sCr^Δ^ criterion (in the first week of life); (3) preceded by critical illness and inversely proportional to GA and BW; (4) had a negligible clinical contribution to death in predictive modeling; and (5) rarely associated with documentation, renal imaging, or pediatric nephrology inpatient consults and outpatient evaluation.

In adults and children, including term neonates, AKI may lead to life-threatening edema, multiorgan system dysfunction, and death that can be attenuated via kidney replacement therapy.^1,2,10^ Deaths in neonates, and in this specific cohort, were associated with substantial respiratory, cardiovascular, and hematologic dysfunction as measured by the nSOFA and VIS; mitigation of AKI does not reduce the incidence of deaths in this unique population.^12,13,19^ Multivariable logistic regression can reveal changes in risk of an outcome, but does not inform whether the variable risk is linear or whether there is an even contribution to the outcome. SHAP-based analysis and visualization of clinical variables that convey the importance of each variable as a singular entity and relative to competing risks may improve identification of key factors. Several lines of evidence in this study suggested AKI, although associated with death as seen in other studies,^6,7,8,9^ was not a major contributor to death. Rather, AKI was commonly a consequence of antecedent organ dysfunction, including shock and respiratory failure as captured by the VIS and nSOFA, rather than a promulgating edema that caused organ dysfunction other than the kidney that could be mitigated by kidney replacement therapy as in pediatric intensive care unit cohorts.^22,23^ In our cohort, most patients who died never manifested AKI as defined by KDIGO criteria. This observation is unlikely to be the result of inadequate measures of UOP or sCr concentrations. A lack of AKI was likely influenced by the time from event to death, which can be rapid. Because severe critical illness may be present at birth due to physiologic immaturity, inclusion of the predicted AKI amelioration with the outcome of death, as well as a temporal measure of kidney replacement therapy feasibility, might improve future studies of AKI in ELBW infants.

Consistent with prior studies, AKI was common (44%) and heavily concentrated in the smallest and least mature infants in our cohort.^5,24,25^ Our incidence of sAKI was higher (24%) compared with Hingorani et al^5^ (18%) and Askenazi et al^24^ (19%), which may reflect the use of 2 unique versions of a modified KDIGO definition on the same cohort of patients. These studies limited the population to 24 to less than 28 weeks’ GA, whereas we included all inborn neonates less than 29 weeks’ GA, including 63 infants at 23 weeks’ GA or less. We included all sCr measures in patients who survived 12 hours or longer, whereas these studies of the same cohort excluded patients who died within 3 days of birth and excluded sCr values measured prior to postnatal day 3. Neither of these studies included UOP as part of the KDIGO definition and Hingorani et al^5^ further modified the KDIGO definition to exclude any reference sCr concentration less than or equal to 0.5 mg/dL. Among infants with AKI in the first week of life in our cohort, most met AKI criteria due to sCr^Δ^ (88%) rather than oliguria (16%). A lack of AKI recognition, defined largely by sCr^Δ^, likely contributed to the low frequency of sAKI documentation (34 of 105 [32%]), imaging (48 [46%]), specialty consultation (17 of 105 [16%]), and specialty/follow-up (6 of 64 [9%]), as noted with the results in other NICU cohorts.^6,26^ Thirty-seven percent of patients with sAKI after the first week of life experienced an sCr^Δ^ to a value less than 1 mg/dL, indicating the reference sCr concentration was less than 0.5 mg/dL. Exclusion of reference sCr values less than or equal to 0.5 by Hingorani et al^5^ may reflect the view that sCr^Δ^ greater than or equal to 2-fold from these values, although meeting the criteria for KDIGO-defined sAKI, may not lead to poor outcomes and warrant AKI-driven practice changes, including pharmacokinetics consultation for medication adjustment, kidney imaging, nephrology consultation, and outpatient follow up.^4,5,6,24,27^ We assembled sCr values and trajectories by DOL among survivors in our cohort who did not have AKI (eFigure 10, eTable 5 in the Supplement). Documentation of AKI duration and criteria met should be included in future studies and would be expected to inform the risk of AKI-related complications in early childhood.^27^ Development, validation, and use of a neonatal AKI definition that is not uniquely modified in each study would improve the generalizability of the results and facilitate clinician recognition. Automated electronic health record–embedded severity of illness metrics (nSOFA, VIS) may improve AKI recognition and reduce AKI-associated complications by increasing nephrology engagement, including relevant imaging and nephrotoxic drug monitoring.^28,29,30,31^

### Limitations

This study has limitations inherent to all single-center, retrospective, discovery-oriented analyses. Although our experience will align with other centers, we acknowledge these retrospective data cannot inform care. To our knowledge, this is the largest cohort of inborn, less than 29 weeks’ GA, ELBW infants in whom the incidence and timing of AKI were measured from birth to death or discharge in the context of severity of illness measures, using data from clinical practice in the context of the published KDIGO definition without additional modifications and with minimal patient exclusions. Outborn ELBW infants were not included and results from that population may be different due to a greater risk of adverse outcomes than noted with inborn infants.^32,33,34^ Institution-specific practices, including use of nephrotoxic medications, may modify both the severity of illness metrics and AKI frequency and affect the generalizability of our results.

## Conclusions

In this cohort study of ELBW infants, AKI was common, occurred early after birth, was inversely associated with gestational age, and was associated with antecedent critical illness and the use of vasoactive-inotropic drugs. Acute kidney injury was associated with but not clearly a major contributor to death.

## Notes

Supplement. **eMethods.** Supplemental Definitions and Analytical Methods **eFigure 1.** CONSORT Diagram **eFigure 2.** Patients With Creatinine and UOP Measures in First Week of Life **eFigure 3.** First-Week Acute Kidney Injury (AKI) in the Extremely Low Birth Weight, Extremely Preterm Infant **eFigure 4.** Creatinine, UOP, and Net Fluid Balance Among Those With and Without First-Week Acute Kidney Injury (AKI) **eTable 1.** UOP (mL/kg/h) by Day of Life by AKI Designation **eFigure 5.** Heat Maps of Individual Patient Acute Kidney Injury (AKI) Trajectory in the First Week of Life by Birth Weight and Gestational Age Group **eFigure 6.** Trajectory Among Infants Who Survived to DOL8 by First Week of Life—AKI Status **eTable 2.** Odds Ratios for sAKI ≤DOL7 by Disease **eTable 3.** Odds Ratios for Death ≤DOL7 **eFigure 7.** Structural Models and SHAP Values **eFigure 8.** Histograms of Logistic Regression Model Coefficients for Death (Anytime) **eTable 4.** Diagnoses Among All Deaths **eFigure 9.** Timing of Medication Doses and Acute Kidney Injury (AKI) During the Hospitalization **eFigure 10.** Daily Creatinine Values and Trajectory Among ELBWS Without Any Acute Kidney Injury (AKI) During the Hospitalization **eTable 5.** Daily Serum Creatinine Among ELBW, <29-Week Infants With No AKI **eTable 6.** AKI During Hospitalization by Gestational Age Group
eReferences
