# Supplementary material for: Frequency of Acute Kidney Injury and Association With Mortality Among Extremely Preterm Infants
Source: JAMA Netw Open. 2022 Dec 13;5(12):e2246327. doi: 10.1001/jamanetworkopen.2022.46327 (PMC9856227; doi:10.1001/jamanetworkopen.2022.46327)
Supplement: Supplement. — eMethods. Supplemental Definitions and Analytical Methods eFigure 1. CONSORT Diagram eFigure 2. Patients With Creatinine and UOP Measures in First Week of Life eFigure 3. First-Week Acute Kidney Injury (AKI) in the Extremely Low Birth Weight, Extremely Preterm Infant eFigure 4. Creatinine, UOP, and Net Fluid Balance Among Those With and Without First-Week Acute Kidney Injury (AKI) eTable 1. UOP (mL/kg/h) by Day of Life by AKI Designation eFigure 5. Heat Maps of Individual Patient Acute Kidney Injury (AKI) Trajectory in the First Week of Life by Birth Weight and Gestational Age Group eFigure 6. Trajectory Among Infants Who Survived to DOL8 by First Week of Life—AKI Status eTable 2. Odds Ratios for sAKI ≤DOL7 by Disease eTable 3. Odds Ratios for Death ≤DOL7 eFigure 7. Structural Models and SHAP Values eFigure 8. Histograms of Logistic Regression Model Coefficients for Death (Anytime) eTable 4. Diagnoses Among All Deaths eFigure 9. Timing of Medication Doses and Acute Kidney Injury (AKI) During the Hospitalization eFigure 10. Daily Creatinine Values and Trajectory Among ELBWS Without Any Acute Kidney Injury (AKI) During the Hospitalization eTable 5. Daily Serum Creatinine Among ELBW, <29-Week Infants With No AKI eTable 6. AKI During Hospitalization by Gestational Age Group eReferences [file jamanetwopen-e2246327-s001.pdf]

Supplementary Online Content

Aziz KB, Schles EM, Makker K, Wynn JL. Frequency of acute kidney injury and association with mortality among extremely preterm infants. *JAMA Netw Open*. 2022;5(12):e2246327. doi:10.1001/jamanetworkopen.2022.46327

eMethods. Supplemental Definitions and Analytical Methods

eFigure 1. CONSORT Diagram

eFigure 2. Patients With Creatinine and UOP Measures in First Week of Life

eFigure 3. First-Week Acute Kidney Injury (AKI) in the Extremely Low Birth Weight, Extremely Preterm Infant

eFigure 4. Creatinine, UOP, and Net Fluid Balance Among Those With and Without First-Week Acute Kidney Injury (AKI)

eTable 1. UOP (mL/kg/h) by Day of Life by AKI Designation

eFigure 5. Heat Maps of Individual Patient Acute Kidney Injury (AKI) Trajectory in the First Week of Life by Birth Weight and Gestational Age Group

eFigure 6. Trajectory Among Infants Who Survived to DOL8 by First Week of Life—AKI Status

eTable 2. Odds Ratios for sAKI ≤DOL7 by Disease

eTable 3. Odds Ratios for Death ≤DOL7

eFigure 7. Structural Models and SHAP Values

eFigure 8. Histograms of Logistic Regression Model Coefficients for Death (Anytime)

eTable 4. Diagnoses Among All Deaths

eFigure 9. Timing of Medication Doses and Acute Kidney Injury (AKI) During the Hospitalization

eFigure 10. Daily Creatinine Values and Trajectory Among ELBWS Without Any Acute Kidney Injury (AKI) During the Hospitalization

eTable 5. Daily Serum Creatinine Among ELBW, <29-Week Infants With No AKI

eTable 6. AKI During Hospitalization by Gestational Age Group

eReferences

This supplementary material has been provided by the authors to give readers additional information about their work.

© 2022 Aziz KB et al. *JAMA Network Open.*

eMethods. Supplemental definitions and analytical methods

Clinical definitions

Pregnancy-induced hypertension (PIH) was defined as a maternal diagnosis of pre-eclampsia or gestational hypertension. Preterm prolonged rupture of membranes (PPROM) was defined as >18 hours before preterm delivery. Preterm labor (PTL) was labor before 37 weeks. Antenatal steroids were defined as the receipt of steroids at any time prior to delivery. Chorioamnionitis was defined as histologic evidence of chorioamnionitis or funisitis. Small for gestational age was defined as <10th percentile for gestational age[1]. Prolonged early antibiotics was ≥5 days of parenteral broad-spectrum antimicrobial treatment started in the first 3 days of life. Spontaneous intestinal perforation (SIP) was intestinal perforation without evidence of necrotizing enterocolitis (NEC). Sepsis was defined as bacteremia stratified by the timing of onset after birth (early-onset: ≤3 days of life; late-onset >3 days of life). Severe intraventricular hemorrhage (SIVH) was defined as grade 3-4 IVH[2]. NEC was defined as modified Bell's stage ≥2[3]. Bronchopulmonary dysplasia (BPD) was defined by the need for respiratory support at 36 weeks' postmenstrual age[4]. Severe retinopathy of prematurity (SROP) was unilateral or bilateral ROP in infants who received laser therapy or bevacizumab in at least 1 eye prior to discharge.

Structural and logistic regression models were built to assess the interactions between the dependent variable and independent variables. Outcomes for regression analysis were death, death ≤7DOL, death >7DOL. We first used univariable linear regression models to assess the association of several factors like sex, race, GA, maximum VIS, nSOFA, sIVH, SIP, NEC, any AKI, and severe AKI (sAKI), and then adjusted for GA, or maximum nSOFA over the first 28 days of life, or maximum VIS with our outcomes. We also used univariable regression for any AKI, sAKI (anytime) and sAKI (≤7DOL and >7DOL). Factors included in this regression were sex, race, GA, maximum VIS^{max}, maximum nSOFA over the first 28 days of life, SIVH, SIP, NEC, and LOS. We subsequently adjusted the AKI related outcomes for GA, maximum nSOFA over the first 28 days of life and maximum VIS.

We used multiple stepwise regression analysis to select factors associated with AKI levels, including some that had reached statistical significance (entry criteria: p value of the F test < 0.05; removal criteria: p value of

© 2022 Aziz KB et al. *JAMA Network Open.*

the F test > 0.10) and some based on clinical relevance and known literature. Stata 15.2 (StataCorp) was used to conduct regression analyses. We considered a 2-sided P-value < 0.05 as statistically significant.

A structural Bayesian model and a logistic regression model were built to assess the interactions between the dependent variable and independent variables. Specifically, for the structural model the Chow-Liu tree algorithm implemented in the 'bnlearn' python package was used to maximize the most likely directed acyclic graph structure followed by an independence test to prune any redundant node edge pairs. For logistic regression 'sci-kit learn's' implementation was used with some default parameters and the following set parameters: penalty='l2', C=1e-2, max_iter=1000, dual=False, tol=1e-3. We chose to use penalized logistic regression with a strength of C=1e-2 to help with generalization for out of sample predictions. No feature engineering, normalization or preprocessing was used for any of the features. SHAP values were used to determine which variables were most important to the logistic regression models. All modeling was performed using Python (version 3.7).

© 2022 Aziz KB et al. *JAMA Network Open.*

eFigure 1. CONSORT diagram.

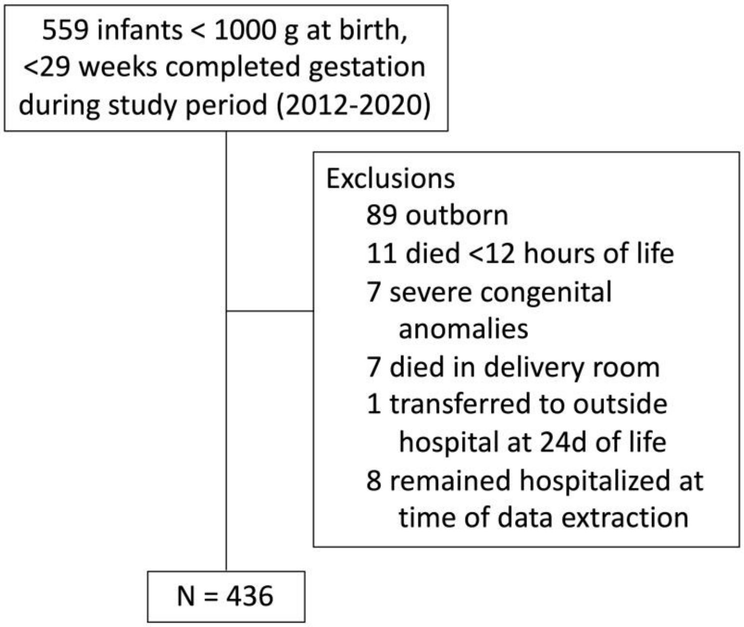

© 2022 Aziz KB et al. *JAMA Network Open.*

eFigure 2. Patients with creatinine and UOP measures in first week of life. There were 5144 measures of Cr in 436 subjects in the first week of life (DOL1: n=432, DOL2: 423, DOL3: 400, DOL4: 386, DOL5: 362, DOL6: 343, DOL7: 318). UOP was recorded on each day alive for 436 subjects (DOL1: n=305 (*only UOP measures for neonates ≥8 hours old*), DOL2: 425, DOL3: 403, DOL4: 395, DOL5: 388, DOL6: 385, DOL7: 384).

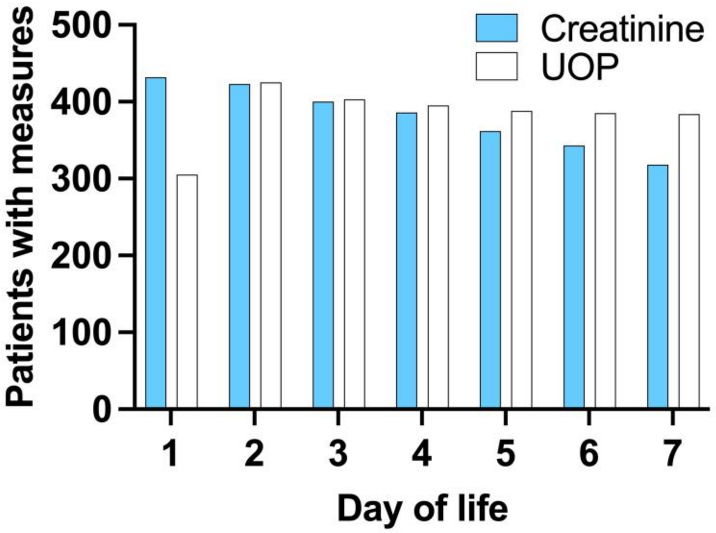

© 2022 Aziz KB et al. *JAMA Network Open.*

eFigure 3. First-week acute kidney injury (AKI) in the extremely low birth weight, extremely preterm infant. A. Frequency of AKI by day of life among all patients in the cohort. **B.** Distribution of AKI type by day of life among those with AKI. **C.** *Kidney Disease: Improving Global Outcomes* (KDIGO) criterion met for AKI by day of life among those with AKI.

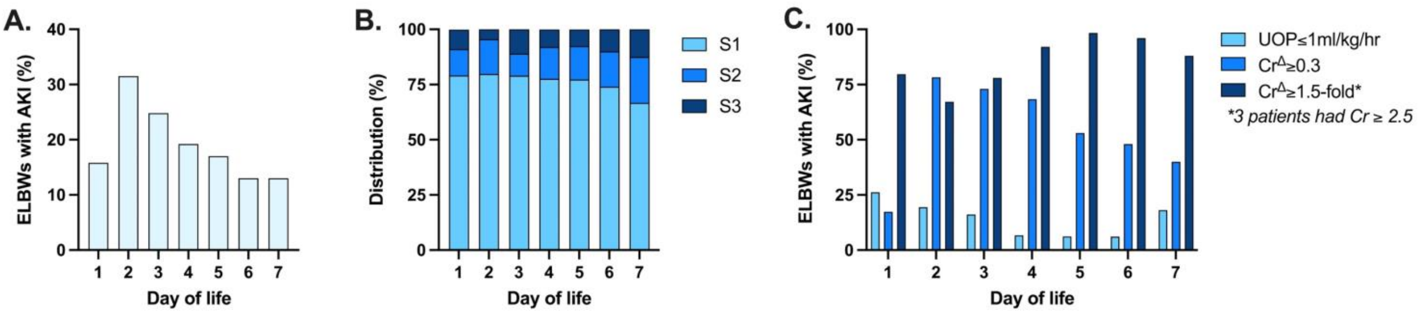

© 2022 Aziz KB et al. *JAMA Network Open.*

eFigure 4. Creatinine, UOP, and net fluid balance among those with and without first-week acute kidney injury (AKI). A. Maximum creatinine values by day of life for patients with and without AKI. Medians and interquartile ranges are shown. **B.** Urine output (mL/kg/hour) values by day of life for patients with and without AKI. Medians and interquartile ranges are shown. **C.** Net fluid balance values by day of life for patients with and without AKI. Medians and interquartile ranges are shown.

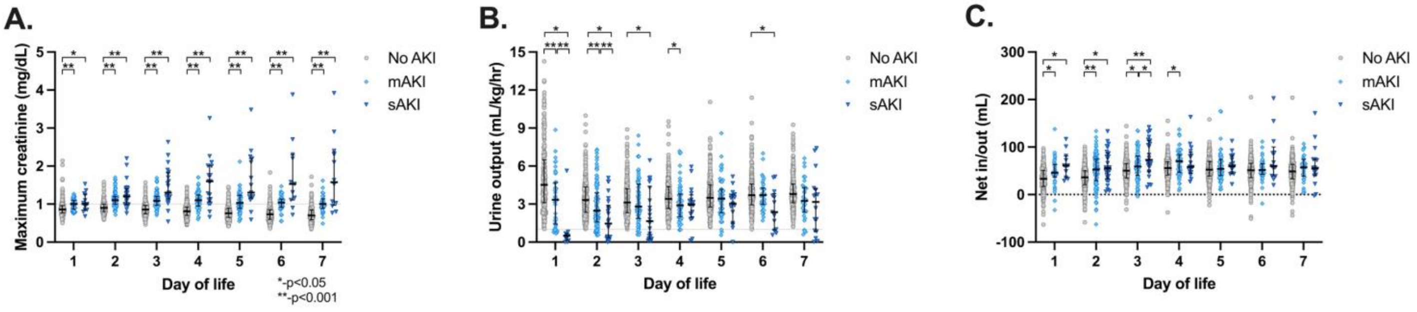

© 2022 Aziz KB et al. *JAMA Network Open.*

eTable 1. UOP (mL/kg/h) by day of life by AKI designation

Group	DOL	25th percentile	50th percentile	75th percentile
**No AKI**	1	3.122	4.515	6.502
	2	2.36	3.32	4.361
	3	2.315	3.13	4.205
	4	2.64	3.41	4.375
	5	2.773	3.49	4.52
	6	2.94	3.71	4.60
	7	3.10	3.79	4.61
**Mild AKI**	1	1.42	3.35	4.711
	2	1.593	2.48	3.887
	3	1.863	2.81	4.585
	4	2.01	2.91	3.8
	5	2.33	3.43	4.17
	6	2.98	3.71	4.27
	7	2.385	3.26	4.305
**Severe AKI**	1	0.1415	0.5019	0.6756
	2	0.355	1.463	2.885
	3	0.2675	1.655	4.403
	4	1.968	2.95	3.73
	5	1.5	3.04	3.77
	6	0.955	2.37	3.825
	7	0.855	3.17	4.218

© 2022 Aziz KB et al. *JAMA Network Open.*

eFigure 5. Heat maps of individual patient acute kidney injury (AKI) trajectory in the first week of life by birth weight and gestational age group. Each row represents a patient over the first week of life. Each cell represents a day and shows the maximum stage of AKI on that day. Cells shown in black (   ) signify the infant was not aliv[redacted] that day.

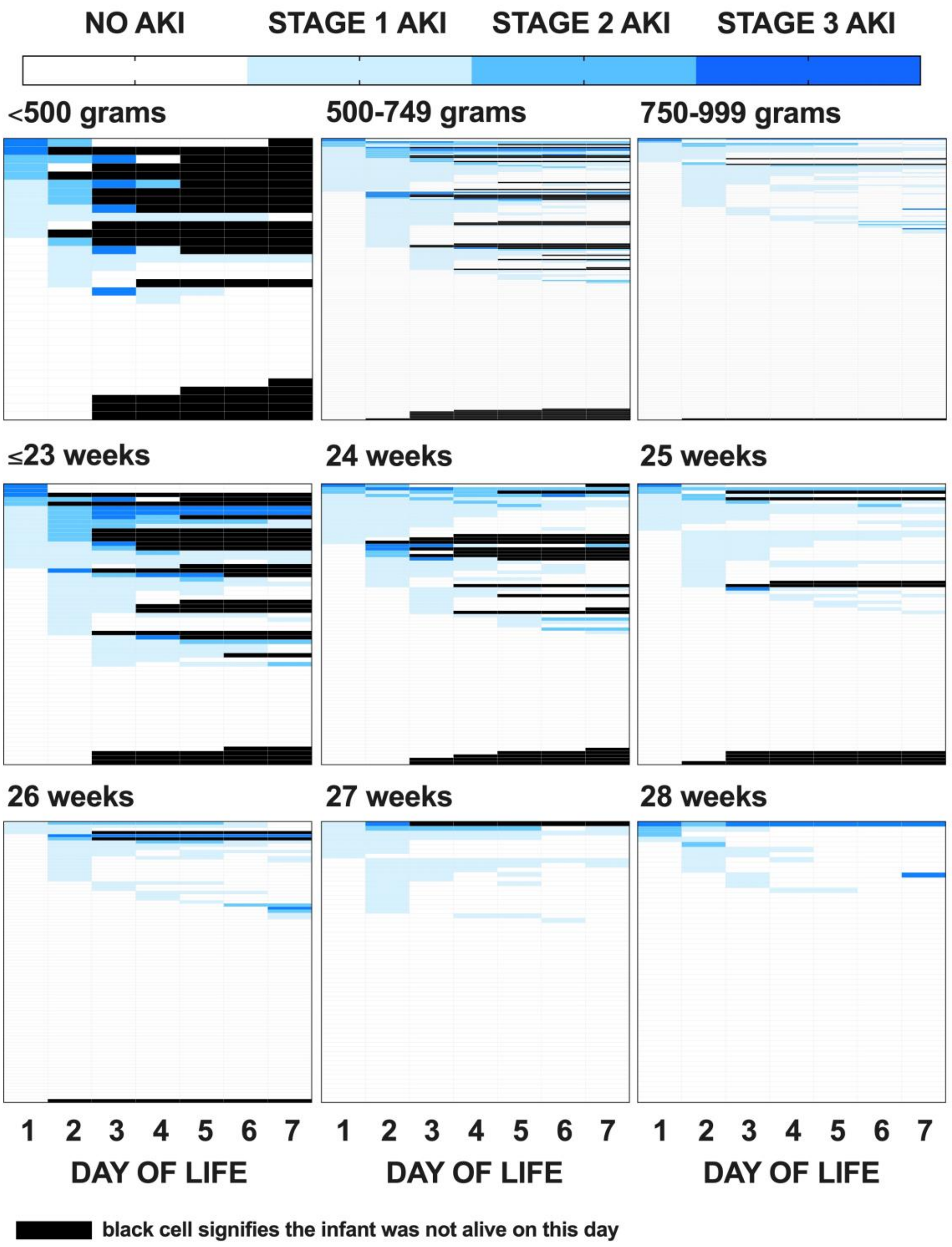

© 2022 Aziz KB et al. *JAMA Network Open.*

eFigure 6. Trajectory among infants who survived to DOL8 by first week of life—AKI status. Among the 229 infants that survived without AKI in the first week, mild AKI developed in 46 (20%; including 5 deaths), severe AKI developed in 22 (10%; including 1 death), while the majority (n=161; 70%; including 6 deaths) never manifested AKI. Among the 116 surviving infants with mild AKI in the first week, subsequent death occurred in 12 (10%), mild AKI reoccurred in 32 (28%; including 2 deaths), severe AKI developed in 25 (22%; including 5 deaths), while 59 (51%; including 5 deaths) never again manifested AKI. In contrast, among the 33 surviving infants with severe AKI in the first week, mild AKI developed in 6 (18%; including 3 deaths), severe AKI reoccurred in 12 (36%; including 4 deaths), and 15 (46%; including 3 deaths) had no further episodes of AKI. Among the 59 patients that survived to DOL8 and manifested severe AKI at any time during the NICU stay, 47 (80%) experienced the first episode of severe AKI after the first week of life, and 10 (17%) died. More than 1 episode of severe AKI occurred in 28/59 (47%). Antecedent vasoactive-inotropic drug exposure occurred in 44 (75%) and late-onset sepsis or NEC occurred in 30 (51%). Peak creatinine values in severe AKI episodes after the first week of life were < 1 mg/dL in 22/59 (37%; median 1.3; IQR 0.77-1.83).

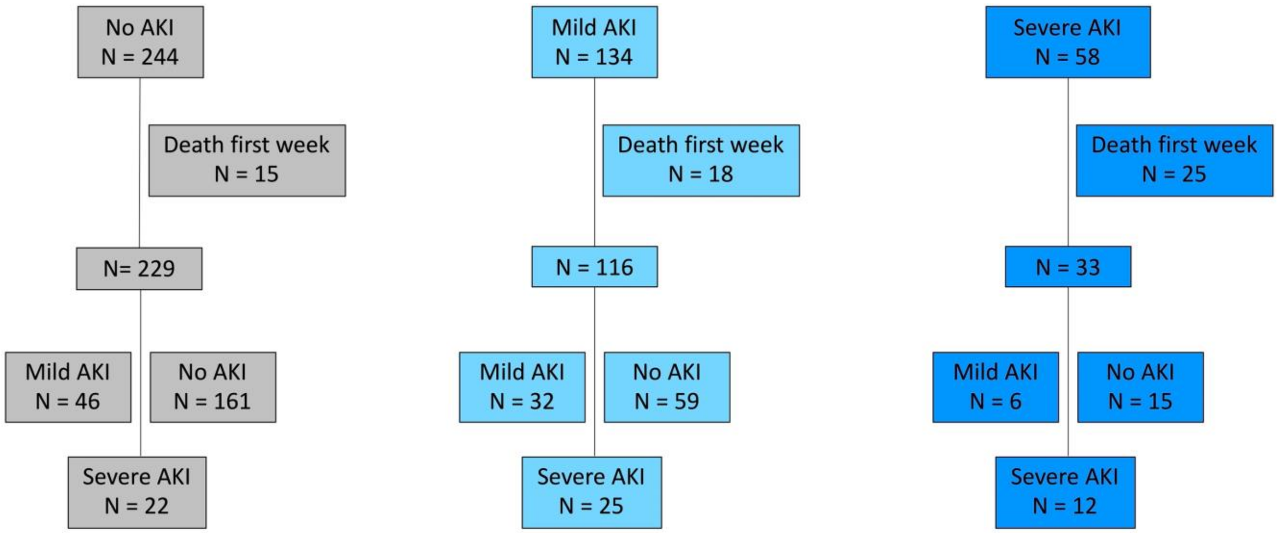

© 2022 Aziz KB et al. *JAMA Network Open.*

eTable 2. Odds ratios for sAKI ≤DOL7 by disease

Variable	OR	p-value	Adjusted for GA	Adjusted for nSOFA$^{max≤DOL7}$	Adjusted for VIS$^{max≤DOL7}$
Sex (male ref)	1.12 (0.63, 1.92)	0.74	1.04 (0.59, 1.85), 0.89	1.22 (0.68, 2.21), 0.51	1.19 (0.66, 2.15), 0.57
Non-white race (white ref)	0.91 (0.49, 1.6)	0.77	1.02 (0.54, 1.9), 0.95	0.84 (0.44, 1.61), 0.62	0.96 (0.51, 1.85), 0.92
GA	0.61 (0.50, 0.74)	<0.001		0.78 (0.62, 0.96), 0.02	0.72 (058, 0.89), 0.002
VIS$^{max≤DOL7}$	1.05 (1.03, 1.07)	<0.001	1.04 (1.02, 1.06), <0.001		
nSOFA$^{max≤DOL7}$	1.27 (1.18, 1.37)	<0.001	1.22 (1.13, 1.33), <0.001		
SIVH	3.73 (2.04, 6.80)	<0.001	2.30 (1.19, 4.47), 0.01	1.95 (1.01, 3.78), 0.047	2.42 (1.24, 4.70), 0.009
SIP	1.70 (0.66, 4.30)	0.27	1.07 (0.40, 2.83), 0.90	1.11 (0.42, 2.95), 0.84	1.46 (0.54, 3.94), 0.45

GA – gestational age, VIS – vasoactive-inotropic score, nSOFA – neonatal sequential organ failure assessment. SIVH – severe (Grade 3-4) intraventricular hemorrhage, SIP – spontaneous intestinal perforation

© 2022 Aziz KB et al. *JAMA Network Open.*

eTable 3. Odds ratios for death ≤DOL7

Variable	OR	p-value	Adjusted for GA	Adjusted for any AKI$^{\leq DOL7}$	Adjusted for any sAKI$^{\leq DOL7}$
Sex (male ref)	0.74 (0.42, 1.28)	0.28	0.61 (0.32, 1.13), 0.12	0.69 (0.39, 1.23), 0.22	0.68 (0.37, 1.23), 0.21
Non-white race (white ref)	1.37 (0.76, 2.46)	0.29	1.86 (0.95, 3.6), 0.07	1.4 (0.77, 2.59), 0.26	1.48 (0.79, 2.77), 0.22
GA	0.41 (0.31, 0.52)	<0.001		0.43 (0.33, 0.56), <0.001	0.45 (0.34, 0.58), <0.001
VIS$^{max\leq DOL7}$	1.15 (1.12, 1.19)	<0.001	1.13 (1.09, 1.16), <0.001	1.14 (1.11, 1.18), <0.001	1.14 (1.10, 1.18), <0.001
nSOFA$^{max\leq DOL7}$	1.72 (1.52, 1.96)	<0.001	1.64 (1.43, 1.89), <0.001	1.68 (1.47, 1.91), <0.001	1.66 (1.45, 1.89), <0.001
SIVH	11.15 (5.74, 21.65)	<0.001	5.17 (2.51, 10.67), <0.001	9.66 (4.89, 19.10),<0.001	9.10 (4.50,18.42),<0.001
SIP	0.45 (0.10, 1.93)	0.28	0.18 (0.04, 0.80), 0.03	0.34 (0.07, 1.50), 0.16	0.32 (0.07, 1.47), 0.14
Any AKI$^{\leq DOL7}$	4.41 (2.36, 8.21)	<0.001	2.91 (1.49, 5.67), 0.002		
Severe AKI$^{\leq DOL7}$	7.92 (4.21, 14.88)	<0.001	4.67 (2.31, 9.44), <0.001		

GA – gestational age, VIS – vasoactive-inotropic score, nSOFA – neonatal sequential organ failure assessment. SIVH – severe (Grade 3-4) intraventricular hemorrhage, SIP – spontaneous intestinal perforation, AKI – acute kidney injury, sAKI – severe acute kidney injury.

© 2022 Aziz KB et al. *JAMA Network Open.*

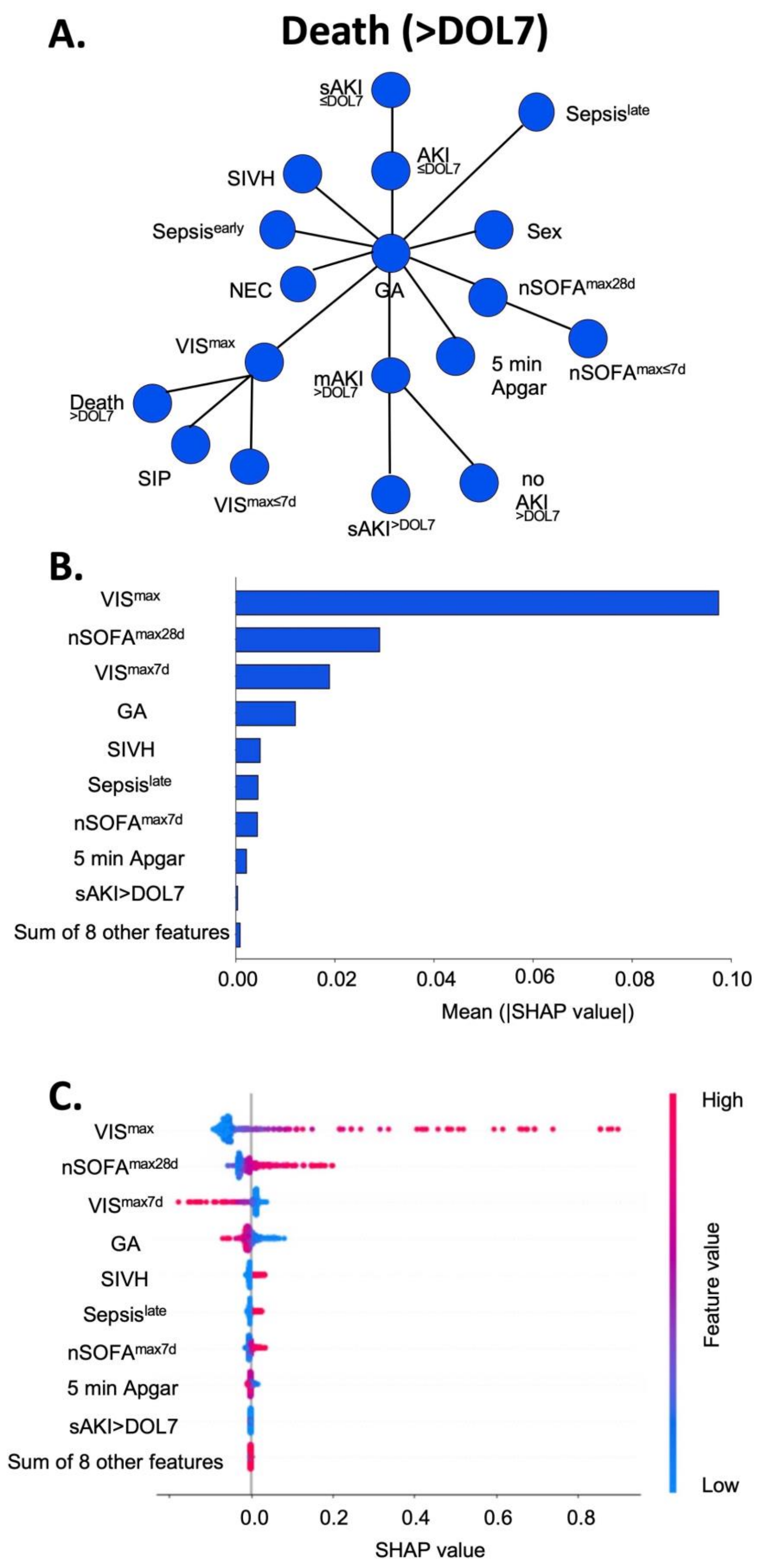

eFigure 7. Structural Models and SHAP Values. A. Structural model for death (>DOL 7) as the primary node. Structural models illustrate relationships between individual clinical variables/outcomes (features) and death (primary node). Lines and circles represent undirected relationships among variables using the maximum likelihood estimation. **B.** SHAP feature importance for death (>DOL 7) as mean absolute SHAP values. **C.** SHAP summary plot for death (>DOL 7)**.** SHAP feature importance and summary plots visually represent the importance of individual variables, effects on the model, and directionality of effect. Sum of 8 other features includes sex, spontaneous intestinal population (SIP), necrotizing enterocolitis (NEC), early onset sepsis, AKI ≤DOL7, sAKI ≤DOL7, mAKI >DOL7, and no AKI >DOL7.

© 2022 Aziz KB et al. *JAMA Network Open.*

eFigure 8. Histograms of logistic regression model coefficients for death (anytime). A. Gestational age (GA) in week. **B.** Apgar score at 5 minutes **C.** 28-day $nSOFA^{max}$ score **D.** VIS^{max} score **E.** Any acute kidney injury (AKI) **F.** severe AKI (sAKI). Note that ‘clustering’/ histogram peaks represent the predominant coefficients. Logistic regression model performance for death at anytime as denoted by weighted average (avg) of **G.** precision, **H.** recall and **I.** f1-score. Note that ‘clustering’/ histogram peaks represent the predominant performance of the logistic regression model.

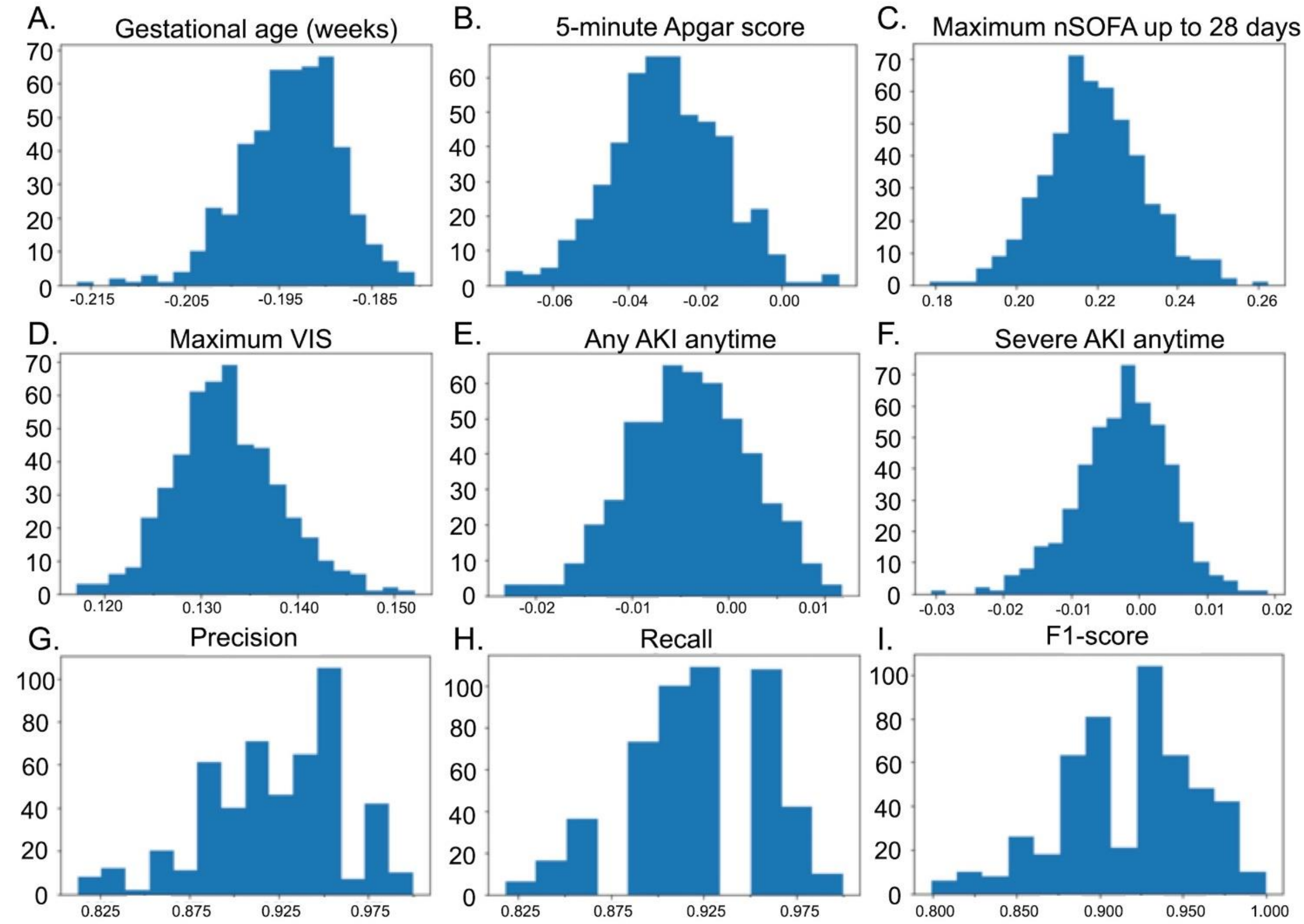

© 2022 Aziz KB et al. *JAMA Network Open.*

eTable 4. Diagnoses among all deaths (n=92)

Diagnoses	N	Sum
SIVH	15	15
shock/respiratory failure	13	28
NEC *totalis*	8	36
respiratory failure	7	43
perforated bowel	5	48
late-onset sepsis	5	53
NEC	4	57
early-onset sepsis/shock/respiratory failure	4	61
SIP/late-onset sepsis	4	65
SIVH/shock	4	69
pneumothorax/respiratory failure	3	72
shock	3	75
shock/respiratory failure/SIVH	3	78
respiratory failure/pulmonary hemorrhage	2	80
pneumonia/shock/respiratory failure	2	82
shock, SIP	2	84
AKI	2	86
cardiac tamponade	1	87
cor pulmonale	1	88
respiratory failure/chronically ill/parents requested redirection of care	1	89
shock/pneumothorax, pulmonary hemorrhage	1	90
shock/early-onset sepsis	1	91
SIVH/SIP	1	92

SIVH – severe (grade 3-4) intraventricular hemorrhage

NEC – necrotizing enterocolitis

SIP – spontaneous intestinal perforation

AKI – acute kidney injury

© 2022 Aziz KB et al. *JAMA Network Open.*

eFigure 9. Timing of medication doses and acute kidney injury (AKI) during the hospitalization. Open circles represent all medication instances and all episodes of creatinine-based AKI-qualifying criterion by day of life. Black bars represent the median. Dotted lines separate classes of medications. Instances of Cr-based severe AKI occurred earlier than mild AKI (median day of life 2; IQR 0, 14 vs. median day of life 5; IQR 3, 21). Although there was definitive temporal overlap between medication dose timing and instances of AKI, ≥75% of vancomycin, amphotericin, chlorothiazide, bumetanide, piperacillin-tazobactam, furosemide, tobramycin, and amikacin doses occurred after 75% of the severe AKI instances had occurred. In contrast, most indomethacin doses occurred prior to a majority of AKI (mild or severe), and most norepinephrine and dopamine exposures occurred prior to most instances of mild AKI.

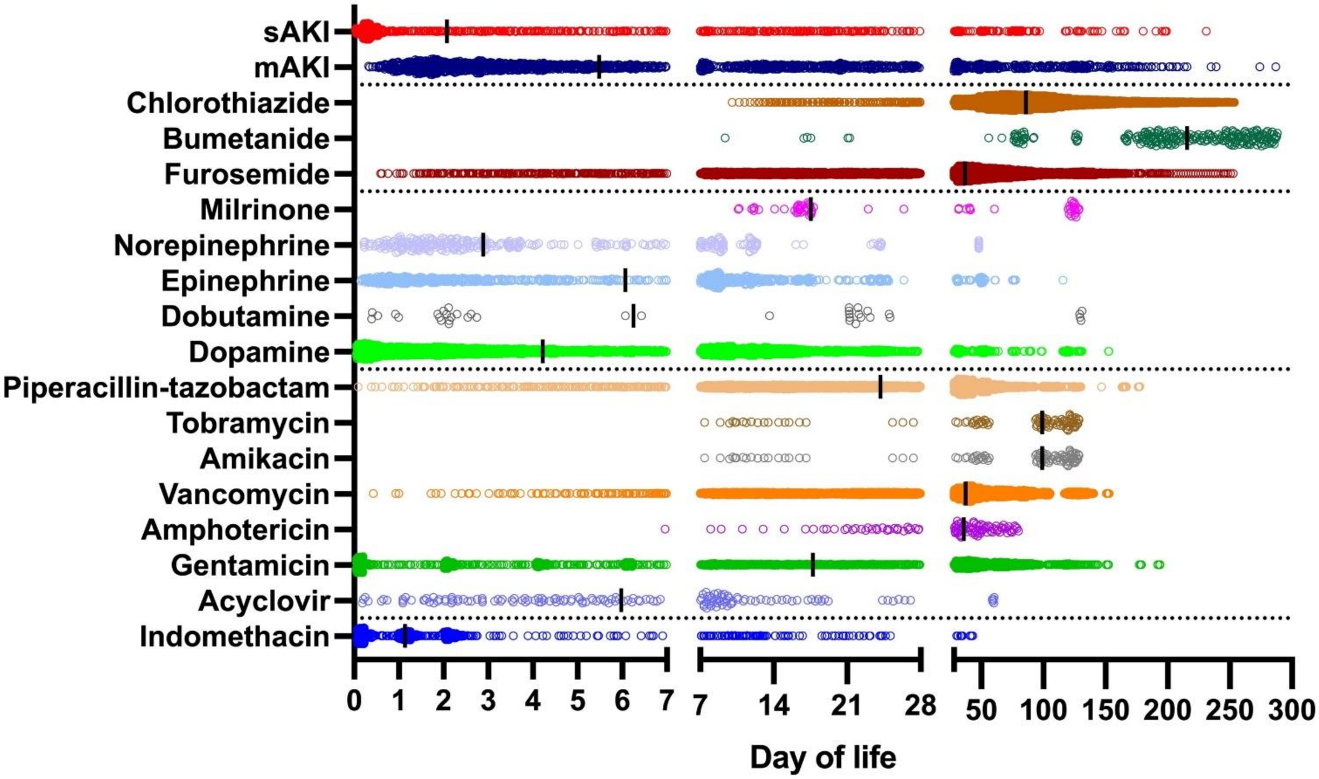

© 2022 Aziz KB et al. *JAMA Network Open.*

eFigure 10. Daily creatinine values and trajectory among ELBWs without any acute kidney injury (AKI) during the hospitalization. A. Creatinine values by day of life (1-60) among survivors that did not meet any criterion for AKI at any time during the hospitalization. **B.** Individual creatinine trajectories for survivors that did not meet any criterion for AKI at any time during the hospitalization. We examined the daily range of 3409 creatinine measurements for the first 60 days of life among the 155 survivors (median GA 26.57; IQR 25.7, 27.57; median BW: 830g, 725, 910) that never met criteria for AKI during the NICU hospitalization and graphed the trajectory of creatinine measures for each of these patients. The 95th percentile for Cr values dropped below 1 mg/dL beginning on DOL16 and was below 0.75 by DOL28.

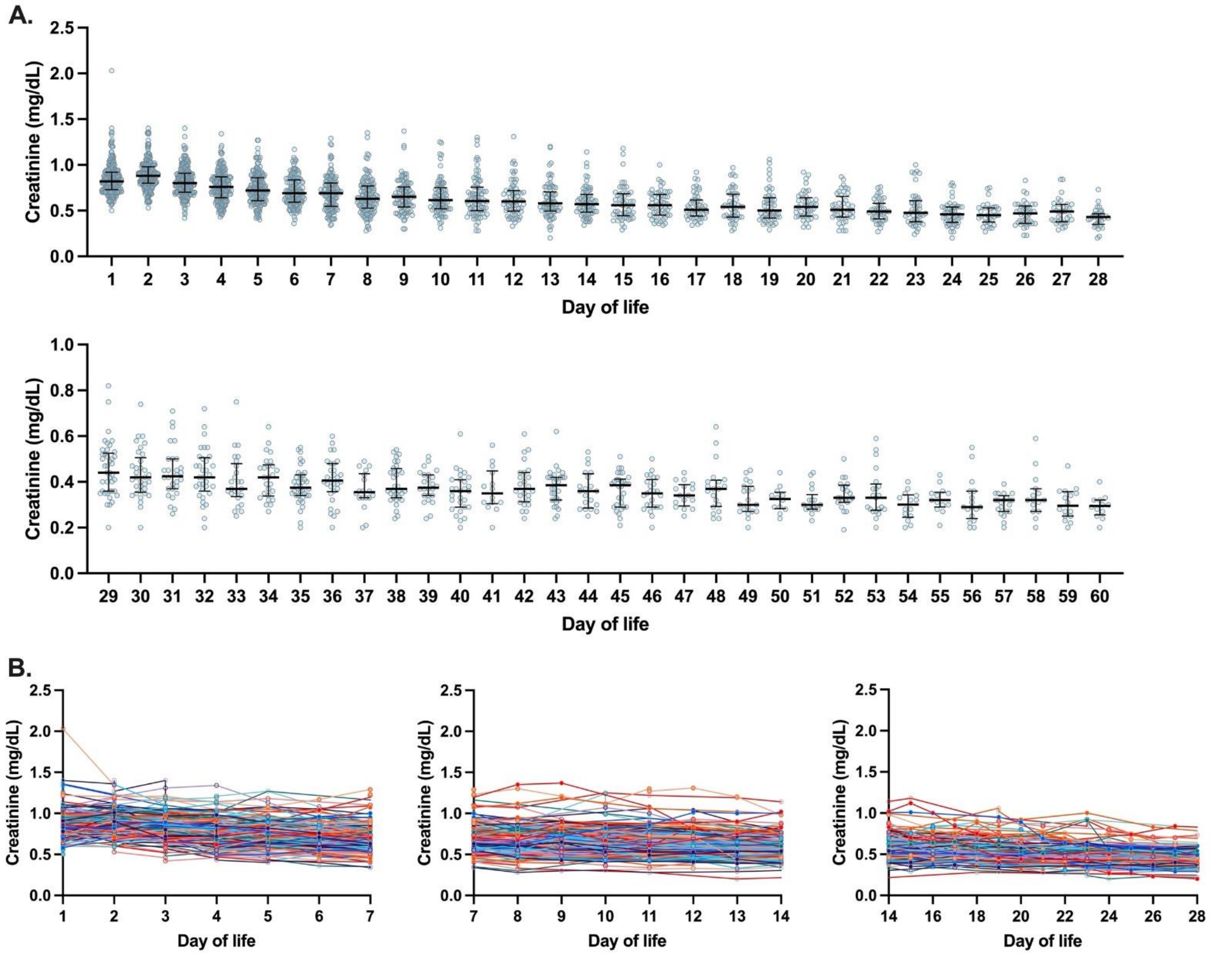

© 2022 Aziz KB et al. *JAMA Network Open.*

eTable 5. Daily serum creatinine among ELBW, <29-week infants with no AKI.

DOL	N	5th percentile	25th percentile	50th percentile	75th percentile	95th percentile
1	292	0.63	0.73	0.82	0.92	1.16
2	278	0.66	0.80	0.88	0.98	1.17
3	256	0.60	0.70	0.80	0.91	1.06
4	211	0.51	0.64	0.76	0.87	1.04
5	169	0.48	0.61	0.72	0.86	1.08
6	144	0.49	0.59	0.69	0.84	1.00
7	130	0.41	0.55	0.69	0.80	1.08
8	122	0.40	0.53	0.63	0.77	1.05
9	90	0.42	0.54	0.65	0.76	0.91
10	78	0.41	0.52	0.62	0.75	1.11
11	80	0.37	0.50	0.61	0.76	1.08
12	65	0.40	0.50	0.60	0.72	1.02
13	73	0.33	0.50	0.58	0.71	1.01
14	66	0.38	0.49	0.57	0.68	0.95
15	53	0.34	0.45	0.56	0.69	1.04
16	56	0.36	0.45	0.56	0.68	0.82
17	49	0.36	0.44	0.51	0.62	0.85
18	48	0.32	0.43	0.54	0.68	0.90
19	51	0.35	0.42	0.50	0.64	0.98
20	43	0.33	0.44	0.54	0.64	0.88
21	44	0.28	0.43	0.51	0.66	0.83
22	43	0.32	0.41	0.49	0.58	0.74
23	46	0.28	0.38	0.48	0.61	0.93
24	48	0.27	0.37	0.46	0.54	0.78
25	33	0.30	0.38	0.45	0.53	0.74
26	41	0.24	0.36	0.47	0.55	0.71
27	34	0.31	0.38	0.49	0.57	0.84
28	33	0.21	0.35	0.43	0.47	0.64
29	41	0.30	0.36	0.44	0.53	0.74
30	33	0.26	0.36	0.42	0.51	0.64
31	28	0.27	0.37	0.43	0.50	0.69
32	33	0.23	0.36	0.42	0.51	0.66
33	25	0.26	0.34	0.37	0.48	0.69
34	26	0.28	0.34	0.42	0.48	0.62

© 2022 Aziz KB et al. *JAMA Network Open.*

35	38	0.22	0.34	0.38	0.43	0.54
36	30	0.23	0.36	0.41	0.48	0.58
37	18	0.20	0.33	0.36	0.44	0.49
38	32	0.25	0.33	0.37	0.46	0.53
39	24	0.24	0.34	0.38	0.43	0.51
40	28	0.21	0.29	0.36	0.41	0.54
41	14	0.20	0.31	0.35	0.45	0.56
42	24	0.25	0.31	0.37	0.44	0.59
43	30	0.25	0.32	0.39	0.42	0.54
44	20	0.25	0.29	0.36	0.44	0.53
45	30	0.23	0.29	0.39	0.41	0.48
46	25	0.22	0.29	0.35	0.41	0.49
47	16	0.25	0.30	0.34	0.39	0.44
48	20	0.24	0.29	0.37	0.41	0.64
49	19	0.20	0.27	0.30	0.38	0.45
50	12	0.24	0.28	0.33	0.36	0.44
51	17	0.23	0.28	0.30	0.35	0.44
52	21	0.20	0.31	0.33	0.39	0.49
53	21	0.20	0.28	0.33	0.39	0.59
54	14	0.20	0.25	0.30	0.34	0.40
55	13	0.21	0.29	0.32	0.36	0.43
56	17	0.20	0.24	0.29	0.36	0.55
57	21	0.20	0.27	0.32	0.34	0.39
58	15	0.20	0.27	0.32	0.37	0.59
59	16	0.20	0.25	0.30	0.36	0.47
60	12	0.20	0.26	0.30	0.32	0.40

© 2022 Aziz KB et al. *JAMA Network Open.*

eTable 6. AKI during hospitalization by gestational age group.

	≤23 (n=63)	24 (n=84)	25 (n=84)	26 (n=89)	27 (n=61)	28 (n=55)	p-value
No AKI (n, %)	13 (20.6)	23 (27.3)	30 (35.7)	42 (47.1)	36 (59.0)	32 (58.1)	<0.001 by X^2
Mild AKI only (n, %)	20 (31.7)	31 (36.9)	39 (46.4)	32 (35.9)	20 (32.7)	13 (23.6)	
Severe AKI (n, %)	30 (47.6)	30 (35.7)	15 (17.8)	15 (16.8)	4 (6.5)	10 (18.1)	

© 2022 Aziz KB et al. *JAMA Network Open.*

eReferences

1. Alexander GR, Himes JH, Kaufman RB, Mor J, Kogan M. A United States national reference for fetal growth. Obstet Gynecol. 1996;87(2):163-168.

2. Papile LA, Burstein J, Burstein R, Koffler H. Incidence and evolution of subependymal and intraventricular hemorrhage: a study of infants with birth weights less than 1,500 gm. J Pediatr. 1978;92(4):529-534.

3. Walsh MC, Kliegman RM. Necrotizing enterocolitis: treatment based on staging criteria. Pediatr Clin North Am. 1986;33(1):179-201.

4. Jensen EA, Dysart K, Gantz MG, et al. The diagnosis of bronchopulmonary dysplasia in very preterm infants. an evidence-based approach. Am J Respir Crit Care Med. 2019;200(6):751-759.

© 2022 Aziz KB et al. *JAMA Network Open.*
